# New endophytic species of *Hypocreales* (*Sordariomycetes*, *Ascomycota*) from the medicinal plant *Panax notoginseng* in China

**DOI:** 10.3897/mycokeys.141.209682

**Published:** 2026-10-06

**Authors:** Shu-Ying Yang, Xiu-Yan Feng, Cui-Yuan Wei, Wen-Bo Zeng, Yuan-Bing Wang, Jian-Ping Li, Zhi-Wei Wang

**Affiliations:** 1 School of Pharmacy, Guizhou University of Traditional Chinese Medicine, Guiyang 550002, China School of Pharmacy, Guizhou University of Traditional Chinese Medicine Guiyang China; 2 State Key Laboratory of Phytochemistry and Natural Medicines, Kunming Institute of Botany, Chinese Academy of Sciences, Kunming 650201, Yunnan, China State Key Laboratory of Phytochemistry and Natural Medicines, Kunming Institute of Botany, Chinese Academy of Sciences Kunming China; 3 Yunnan Key Laboratory for Fungal Diversity and Green Development, Kunming Institute of Botany, Chinese Academy of Sciences, Kunming 650201, Yunnan, China Yunnan Key Laboratory for Fungal Diversity and Green Development, Kunming Institute of Botany, Chinese Academy of Sciences Kunming China; 4 Institute of International Rivers and Eco-Security, Yunnan University, Kunming 650500, Yunnan, China Institute of International Rivers and Eco-Security, Yunnan University Kunming China; 5 Sanqi Medicines College, Wenshan University, Wenshan 663099, Yunnan, China Sanqi Medicines College, Wenshan University Wenshan China

**Keywords:** Endophytic fungi, morphology, new species, *Panax notoginseng*, phylogeny

## Abstract

*Panax notoginseng* is a famous medicinal plant in China. Under forest-based ecological cultivation, its roots harbor a diverse assemblage of endophytic fungi with unique community structures. Some of these fungi can enhance host resistance to diseases and environmental stresses, promote plant growth, and may influence the biosynthesis of medicinal saponins, thereby contributing to host fitness and metabolite regulation. In this study, a total of 188 endophytic fungal isolates were obtained from the roots of *P. notoginseng* cultivated under a forest-based ecological system in Yunnan Province, China, including 70 isolates belonging to *Hypocreales*. Three undescribed taxa of *Hypocreales* were further investigated based on morphological observations and multilocus phylogenetic analyses, resulting in the description of three new species: *Trichoderma wenshanense*, *Clonostachys grandispora*, and *Thelonectria chlamydosporia*. Detailed descriptions, notes, and illustrations are provided for these new species. This study expands the known diversity of endophytic fungi associated with *P. notoginseng* and provides taxonomically characterized fungal resources for future studies on their potential applications in ecological cultivation and medicinal quality improvement.

## Introduction

*Panax notoginseng* (Burk.) F.H. Chen (*Araliaceae*) is a renowned medicinal plant in Yunnan Province, China (Meng et al. 2016). However, traditional cultivation methods often face sustainability challenges due to continuous cropping obstacles and soil-borne diseases. Forest-based ecological cultivation is an important model for sustainable *P. notoginseng* cultivation. Unlike conventional cultivation, the forest-based, semi-wild cultivation environment differs markedly from farmland cultivation systems in light conditions, temperature, humidity, litter composition, and soil physicochemical properties and may therefore harbor endophytic fungal assemblages distinct from those in farmland-grown plants (Allan et al. 2015; Zhang et al. 2019; Zhang et al. 2021; Wang et al. 2023; Li et al. 2024). Under this model, the roots harbor abundant and diverse endophytic fungi (Zheng et al. 2017; Li et al. 2021; Lin et al. 2024). Previous studies have shown that endophytic fungi associated with *P. notoginseng* exhibit high species diversity and include numerous undescribed taxa (Zheng et al. 2017; Lin et al. 2024). In recent years, several new endophytic fungal species have been reported from *P. notoginseng*, including *Trichoderma panacis* S.Y. Liu, T.Y. Zhang, Y. Yu & Y.X. Zhang (Liu et al. 2020), *Verruconis panacis* T. Zhang & Y.X. Zhang (Zhang et al. 2018), *Drechmeria panacis* T.Y. Zhang, and Y. Yu & Y.X. Zhang (Yu et al. 2018), thereby expanding the understanding of the diversity of new fungi associated with this medicinal plant. Therefore, systematic taxonomic studies of endophytic fungi associated with *P. notoginseng* are essential for enriching species diversity and revealing unique fungal taxa associated with medicinal plants. Recent fungal diversity surveys in southwestern China have also demonstrated substantial undiscovered diversity of filamentous and entomopathogenic fungi, emphasizing the importance of continued exploration and taxonomic investigation of fungal resources (Xu et al. 2026).

Previous surveys have revealed high species diversity of endophytic fungi in different tissues of *P. notoginseng*. Zhang and Li (2011) isolated 158 endophytic fungal strains from the roots, stems, and leaves of healthy *P. notoginseng*, with dominant taxa including *Fusarium* Link, *Chaetomium* Kunze, and members of *Xylariaceae* (Liang 2019; Zhang and Li 2011). The number and distribution of endophytic fungi differed significantly among organs. Zheng et al. (2017) isolated 89 endophytic fungal strains from the roots, stems, leaves, and seeds of *P. notoginseng*, representing 23 genera, and also reported an unidentified taxon belonging to *Sordariomycetes*. Li et al. (2021) isolated 24 endophytic fungal strains from *P. notoginseng*, belonging to 12 genera, with *Chaetomium globosum* Kunze as the dominant species in fibrous roots. Both culture-dependent surveys and high-throughput sequencing studies indicated that the roots of *P. notoginseng* still harbored numerous fungal taxa with unresolved taxonomic placements, many of which have not been described. Ambiguous species-level identification and taxonomic uncertainty remain major obstacles to functional exploration and resource utilization (Lin et al. 2024).

Furthermore, many endophytic fungi associated with *P. notoginseng* have shown considerable potential in promoting plant growth, enhancing disease resistance, and mediating the biotransformation of bioactive compounds (Liu et al. 2024). For instance, some endophytic fungi from *P. notoginseng* are capable of synthesizing plant growth regulators such as indole-3-acetic acid and gibberellins, thereby promoting host plant growth (Liu et al. 2024). *Trichoderma gamsii* Samuels & Druzhin. strain YIM PH30019, isolated from *P. notoginseng*, can suppress soil-borne diseases by releasing volatile organic compounds (Chen et al. 2016). Some endophytic fungi can also modulate host metabolic pathways, thereby promoting the accumulation of bioactive saponins in *P. notoginseng*. Specifically, *Fusarium* sp. PN8 is potentially involved in the biosynthesis of ginsenosides Rb1, Rd, and Rg3, whereas *Aspergillus* sp. PN17 may produce key saponin components, including Re, Rd, and Rg3, indicating their potential for improving the medicinal quality of *P. notoginseng* (Jin et al. 2017). *Trichoderma asperellum* Samuels, Lieckf. & Nirenberg strain Ta1, isolated from the rhizosphere soil of *P. notoginseng*, was reported to inhibit the growth of *Fusarium solani* (Mart.) Sacc. and significantly enhance plant growth and saponin accumulation (Gu et al. 2025). Additionally, in other crop systems, the congeneric species *Trichoderma harzianum* Rifai can suppress *Fusarium* diseases by detoxifying fusaric acid and modulating rhizosphere interactions (Cao et al. 2025). These studies indicate that fungi associated with *P. notoginseng*, particularly members of *Hypocreales*, are of considerable importance for both taxonomy and applied research.

In summary, root endophytic fungi of *P. notoginseng* exhibit high species diversity and include numerous undescribed taxa (Zhang and Li 2011; Zheng et al. 2017; Li et al. 2021; Lin et al. 2024; Liu et al. 2024), representing an important potential source for the discovery of new species. However, strains isolated and identified to date constitute only a small fraction of these rich fungal resources (Liu et al. 2024). Consequently, systematic studies of endophytic fungi associated with *P. notoginseng* remain insufficient, and numerous potential new taxa await further discovery and formal description. In this study, endophytic fungi were isolated and purified from root tissues of *P. notoginseng* cultivated under a forest-based ecological system in Wenshan Prefecture, Yunnan Province, China. Species delimitation was based on morphological characteristics and multilocus phylogenetic analyses. Three new species of *Hypocreales* are described, belonging to *Trichoderma (Hypocreaceae)*, *Clonostachys (Bionectriaceae)*, and *Thelonectria (Nectriaceae)*, respectively. This study aims to clarify the taxonomic positions of these isolates and to provide basic taxonomic data for subsequent investigations into endophytic fungal resources associated with *P. notoginseng*.

## Materials and methods

### Specimen collection and fungal isolation

*Panax notoginseng* plants were collected from an organically cultivated area in Wenshan Prefecture, Yunnan Province, China, where an aerial seeding-based ecological cultivation system was applied. Under this cultivation system, seeds of *P. notoginseng* were aerially sown and allowed to germinate and grow in a natural forest environment without artificial intervention. A total of 23 healthy *P. notoginseng* plants were collected for endophytic fungal isolation. During sampling, whole *P. notoginseng* plants were carefully excavated using pre-sterilized digging tools to minimize root damage while retaining the rhizosphere soil attached to the roots. Each plant was placed individually in a sterile sealed sampling bag, and the sampling location, plant number, and collection date were recorded in detail. Samples were kept in a low-temperature storage box and immediately transported to the laboratory for further processing.

Endophytic fungi were isolated from the roots of *P. notoginseng* using a surface sterilization–tissue homogenization method. The roots were first thoroughly rinsed with sterile distilled water to remove adhering rhizosphere soil and other surface debris. Clean fine-root segments were selected and sequentially immersed in 75% (v/v) ethanol for 5–15 s and 3% (v/v) hydrogen peroxide for 5–15 s, with the exposure time adjusted according to the thickness of the root segments. The root segments were subsequently rinsed twice with sterile distilled water to remove residual disinfectants. The surface-sterilized root tissues were aseptically transferred to autoclaved sterile 10 mL centrifuge tubes and thoroughly homogenized using sterile metal grinding rods.

Fungal isolation was performed on selective potato dextrose agar (PDA) supplemented with antibiotics. The PDA medium was prepared using potato extract from 200 g of peeled potato tubers extracted in 1 L of distilled water, 20 g/L dextrose, 20 g/L agar, and distilled water to a final volume of 1 L, followed by autoclaving at 121 °C for 30 min. After cooling to 50–55 °C, penicillin sodium and streptomycin sulfate were aseptically added to final concentrations of 0.96 g/L and 1.00 g/L, respectively. The medium was thoroughly mixed, poured into 90 mm Petri dishes, and allowed to solidify. The plates were used after surface condensation had evaporated. A 100 μL aliquot of root tissue homogenate obtained using the surface sterilization–tissue homogenization method was spread onto antibiotic-supplemented PDA plates and incubated at 25 °C. Plates were examined regularly, and fungal colonies with different morphologies were selected and purified. All fungal strains were preserved in sterile cryovials using two methods: PDA cultures maintained at 4 °C and glycerol stocks (33% glycerol, v/v) containing mycelial plugs with agar stored at –80 °C. Specimens (dried cultures) were deposited in the Cryptogamic Herbarium of the Kunming Institute of Botany, Chinese Academy of Sciences (KUN-HKAS). All strains isolated in this study were deposited in the Kunming Institute of Botany Culture Collection (KUNCC), Chinese Academy of Sciences.

### Morphological observations

Colony characteristics were recorded on potato dextrose agar (PDA), synthetic low-nutrient agar (SNA), and oatmeal agar (OA). Strains stored at 4 °C were inoculated onto the center of plates containing each medium and incubated at 25 °C. Colony characteristics, including colony diameter, color, texture, margin features, pigment production, and growth rate, were observed and recorded after 7 and 14 d of incubation.

For microscopic observations, sterile glass slides were prepared with a small piece of PDA medium, approximately 2–3 mm thick, placed at the center. A small mycelial plug was taken from the margin of an actively growing colony and inoculated onto the center of the PDA block. The slides were then placed in glass Petri dishes lined with sterile filter paper moistened with sterile water to maintain humidity. *Trichoderma* strains were cultured on PDA at 25 °C for 7 d. When sporulation was not observed under conventional incubation conditions, slide cultures of *Clonostachys* and *Thelonectria* strains were re-established and incubated at 25 °C under UVA irradiation using an OSRAM BL UVA lamp (25 W). The cultures were exposed to UVA light for approximately 7 h per day to stimulate sporulation. Sporulation structures were examined after 14 d for *Clonostachys* and 21 d for *Thelonectria*, respectively. Microscopic characteristics of the asexual morph of each strain were observed and recorded using an Olympus BX53 light microscope (Olympus, Tokyo, Japan).

### DNA extraction, PCR amplification, and sequencing

Strains were cultured on PDA for approximately 7 d, and genomic DNA was extracted using a modified cetyltrimethylammonium bromide (CTAB) method (Möller et al. 1992). In this study, sequences of the internal transcribed spacer region (ITS), the large subunit ribosomal RNA gene (LSU), the RNA polymerase II second-largest subunit gene (*rpb2*), the RNA polymerase II largest subunit gene (*rpb1*), the translation elongation factor 1-alpha gene (*tef1*), the actin gene (*act*), and the beta-tubulin gene (*tub2*) were obtained through PCR amplification and Sanger sequencing. The primer pairs used for amplification were ITS5/ITS4 for ITS (White et al. 1990), LR0R/LR5 for LSU (Vilgalys and Hester 1990), RPB1-a/RPB1-c for *rpb1* (Castlebury et al. 2004), RPB2F/RPB2-7cR and RPB2-5F2/RPB2-7cR for *rpb2* in different taxa (Liu et al. 1999; Sung et al. 2007), EF1-728F/TEF1LLRev, EF1-983F/EF1-2218R, and EF728/EF1567 for *tef1* (Jaklitsch et al. 2005; Rehner and Buckley 2005; Carbone and Kohn 1999), T1/Bt2b and T1/T2 for *tub2* (Glass and Donaldson 1995; O’Donnell and Cigelnik 1997), and TRIACT1/TRIACT2 for *act* (Samuels et al. 2006). PCR amplification conditions followed previously described protocols or the original references for the corresponding primers: Ji et al. (2025) for ITS and *tef1* of *Tri. wenshanense*, Shang et al. (2025) for *rpb2* of *Tri. wenshanense*, Liu et al. (1999) and Sung et al. (2007) for *rpb2* of *Clo. grandispora*, Doilom et al. (2014) for LSU of *Clo. grandispora*, Rehner and Buckley (2005) for *tef1* of *Clo. grandispora*, Udayanga et al. (2015) for *tub2* of *Clo. grandispora*, Vilgalys and Hester (1990) for LSU of *The. chlamydosporia*, Carbone and Kohn (1999) for *tef1* of *The. chlamydosporia*, O’Donnell and Cigelnik (1997) for *tub2* of *The. chlamydosporia*, and Samuels et al. (2006) for *act* of *The. chlamydosporia*. PCR products were purified and sequenced by commercial sequencing services.

### Sequence alignment and phylogenetic analyses

The ITS sequences of all strains were aligned using MAFFT (Katoh et al. 2019), and the alignments were manually adjusted in MEGA7 (Kumar et al. 2016). The aligned sequences were then appropriately trimmed to obtain fragments suitable for subsequent molecular identification. The generic placement of each strain was preliminarily inferred from ITS BLAST searches. Preliminary identification indicated that the three putative new species belonged to different families and genera within *Hypocreales*. Therefore, phylogenetic trees were constructed using different combinations of commonly used loci depending on the corresponding taxonomic groups. Reference sequences were selected based on taxonomic relevance, and sequences from type strains were preferentially included whenever available. Following the molecular identification scheme established for *Trichoderma* (Cai and Druzhinina 2021), the concatenated *tef1* and *rpb2* dataset was used for the *Trichoderma* species. For the *Clonostachys* species, a concatenated five-locus dataset comprising ITS, LSU, *rpb2*, *tef1*, and *tub2* was used. For the *Thelonectria* species, a concatenated six-locus dataset comprising ITS, LSU, *rpb1*, *tef1*, *act*, and *tub2* was used. For the newly described species, all loci used in the multilocus phylogenetic analyses were obtained by PCR amplification and sequencing. The GenBank accession numbers of reference sequences for *Trichoderma* (Cai and Druzhinina 2021), *Clonostachys* (Zhao et al. 2023), and *Thelonectria* (Salgado-Salazar et al. 2012; Zeng and Zhuang 2019) were obtained from previous studies. All sequences were downloaded from the NCBI GenBank database.

Maximum likelihood (ML) and Bayesian inference (BI) phylogenetic trees were constructed for the three putative new species and their closely related taxa. ML analyses were performed using IQ-TREE v. 2.2.0 (Minh et al. 2020) under the GTR+I+G substitution model, with 1,000 bootstrap replicates. BI analyses were conducted using MrBayes v. 3.2 (Ronquist et al. 2012). Four Markov chains were run for 10,000,000 generations, with trees sampled every 1,000 generations. The first 25% of sampled trees were discarded as burn-in, and the remaining trees were used to calculate posterior probabilities. *Nectria eustromatica* CBS 121896, *Acremonium alternatum* CBS 407.66, and *N. cinnabarina* CBS 125165 were designated as outgroup taxa for the *Trichoderma*, *Clonostachys*, and *Thelonectria* phylogenetic analyses, respectively. Phylogenetic trees were visualized using FigTree v. 1.4.2. Only ML bootstrap proportions ≥ 70% (ML-BP) and BI posterior probabilities ≥ 0.7 (BI-PP) are shown at the nodes.

## Results

### Phylogenetic analyses

A total of 188 endophytic fungal isolates were obtained from the roots of 23 forest-grown *P. notoginseng* plants. Based on preliminary ITS identification, 70 isolates were assigned to *Hypocreales*. Among these *Hypocreales* isolates, several isolates belonging to *Trichoderma*, *Clonostachys*, and *Thelonectria* were selected for detailed taxonomic analyses. During subsequent examination of the original fungal collection, additional conspecific isolates corresponding to three undescribed taxa were identified and included in the multilocus phylogenetic analyses. The representative strains KUNCC 11820, KUNCC 11821, and KUNCC 11822 were preliminarily assigned to *Trichoderma (Hypocreaceae)*, *Clonostachys (Bionectriaceae)*, and *Thelonectria (Nectriaceae)*, respectively, based on ITS sequence analyses. The additional isolates KUNCC 11829, KUNCC 11830, and KUNCC 11831 of *Trichoderma*; KUNCC 11834 of *Clonostachys*; and KUNCC 11832 and KUNCC 11833 of *Thelonectria* showed identical or highly similar ITS sequences to the corresponding representative strains, suggesting that they represented the same undescribed taxa. These isolates were subsequently incorporated into the multilocus phylogenetic analyses.

**Figure 1.**
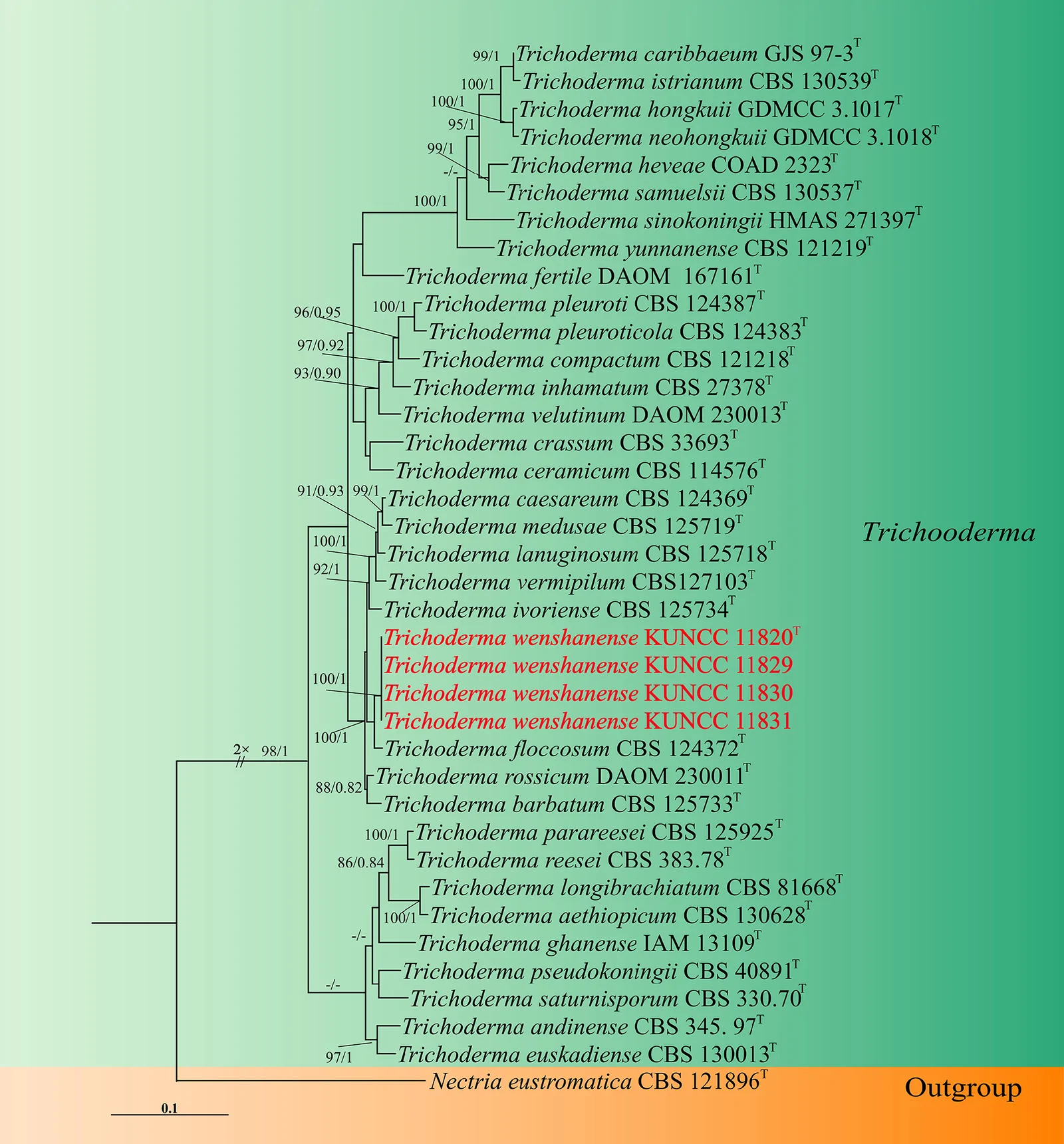
Phylogenetic tree of *Trichoderma* inferred from the concatenated *rpb2* and *tef1* dataset. Maximum likelihood bootstrap proportions ≥ 70% (ML-BP) and Bayesian inference posterior probabilities ≥ 0.7 (BI-PP) are shown at the nodes. *Nectria eustromatica* CBS 121896 was used as the outgroup. Newly described species are highlighted in red, and ex-type strains are indicated by superscript T.

**Figure 2.**
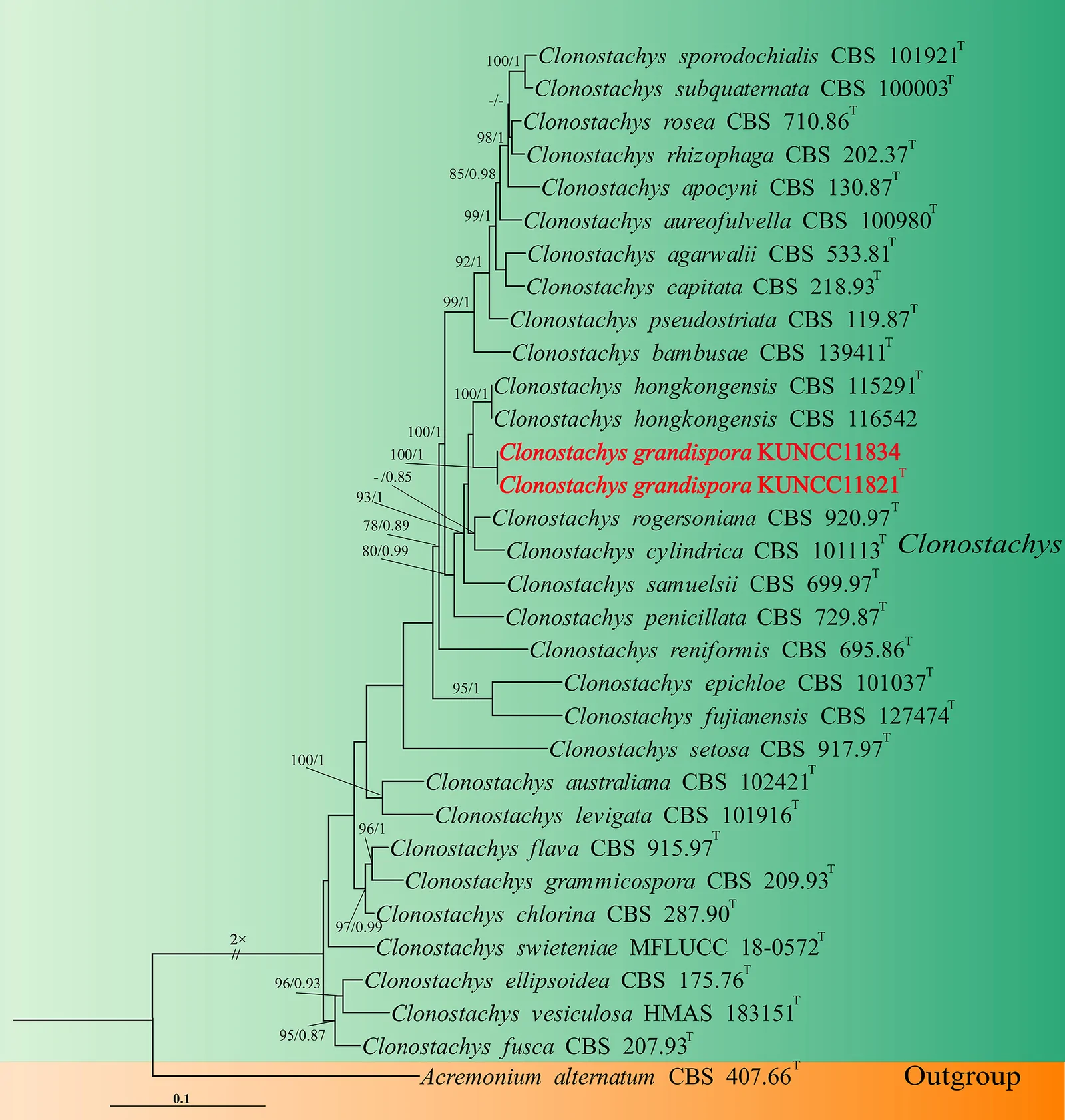
Phylogenetic tree of *Clonostachys* inferred from the concatenated ITS, LSU, *rpb2*, *tef1*, and *tub2* dataset. Maximum likelihood bootstrap proportions ≥ 70% (ML-BP) and Bayesian inference posterior probabilities ≥ 0.7 (BI-PP) are shown at the nodes. *Acremonium alternatum* CBS 407.66 was used as the outgroup. Newly described species are highlighted in red, and ex-type strains are indicated by superscript T.

**Figure 3.**
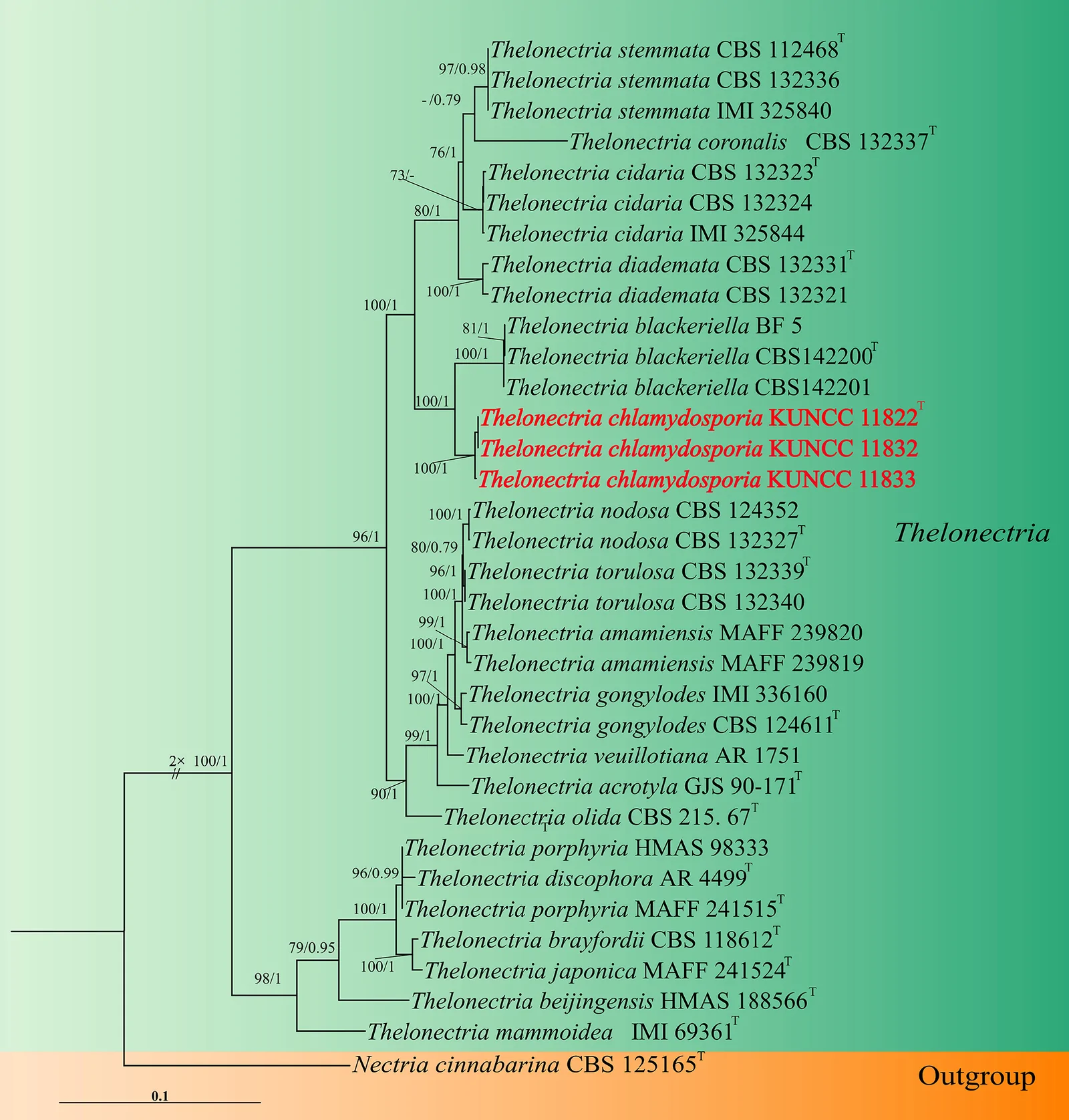
Phylogenetic tree of *Thelonectria* inferred from the concatenated ITS, LSU, *rpb1*, *tef1*, *act*, and *tub2* dataset. Maximum likelihood bootstrap proportions ≥ 70% (ML-BP) and Bayesian inference posterior probabilities ≥ 0.7 (BI-PP) are shown at the nodes. *Nectria cinnabarina* CBS 125165 was used as the outgroup. Newly described species are highlighted in red, and ex-type strains are indicated by superscript T.

To further identify these strains and determine their phylogenetic positions, multilocus phylogenetic analyses were performed separately for each of the three putative new species and their closely related taxa. The ML and BI analyses yielded congruent topologies (Figs 1, 2, 3). For the putative new species of *Trichoderma*, the concatenated length of *tef1* and *rpb2* was 2,159 bp, comprising 1,066 bp of *tef1* and 1,093 bp of *rpb2*. The phylogenetic tree showed that the strains KUNCC 11820, KUNCC 11829, KUNCC 11830, and KUNCC 11831 formed a distinct lineage within *Trichoderma* and were most closely related to *Tri. floccosum* Samuels CBS 124372, with strong support (100/1; Fig. 1). Their position in the multilocus phylogeny supports the recognition of these strains as representing a distinct new species.

For the putative new species of *Clonostachys*, the concatenated ITS, LSU, *rpb2*, *tef1*, and *tub2* dataset comprised 3,784 bp, including 494 bp of ITS, 790 bp of LSU, 736 bp of *rpb2*, 735 bp of *tef1*, and 1,029 bp of *tub2*. In the multilocus phylogeny, KUNCC 11821 and KUNCC 11834 formed a distinct lineage closely related to *Clo. hongkongensis* L. Zhao & Crous (CBS 115291 and CBS 116542), with strong support (100/1; Fig. 2). The examined strains of *Clo. grandispora* differed from *Clo. hongkongensis* strains CBS 115291 and CBS 116542 by 102 and 103 nucleotide sites, respectively, across the concatenated dataset. These molecular differences, together with morphological characteristics, support the recognition of *Clo. grandispora* as a distinct species.

For the putative new species of *Thelonectria*, the concatenated ITS, LSU, *rpb1*, *tef1*, *act*, and *tub2* dataset had a total length of 3,884 bp, comprising 486 bp of ITS, 789 bp of LSU, 624 bp of *rpb1*, 839 bp of *tef1*, 627 bp of *act*, and 519 bp of *tub2*. The phylogenetic tree showed that the strains KUNCC 11822, KUNCC 11832, and KUNCC 11833 formed a distinct monophyletic lineage and were sister to the subclade containing *The. blackeriella* CBS 142200, *The. blackeriella* BF 5, and *The. blackeriella* CBS 142201 (Berk. & M.A. Curtis) Salgado-Salazar & P. Chaverri, with strong support (100/1; Fig. 3), supporting their recognition as a distinct new species.

Taken together, the multilocus phylogenetic results support the recognition of the three taxa as distinct new species. Sequences used in the phylogenetic analyses and their GenBank accession numbers are listed in Suppl. material 1.

### Taxonomy

#### Trichoderma wenshanense

Taxon classificationFungiHypocrealesHypocreaceae

S.Y. Yang, Y.B. Wang, J.P. Li & Z.W. Wang sp. nov.

8883FC75-2230-554D-8B82-FE5DD4AA8A57

Fungal Names: FN 73988

Fig. 4

##### Etymology.

The specific epithet “*wenshanense*” refers to Wenshan Zhuang and Miao Autonomous Prefecture, Yunnan Province, China, where the species was collected.

##### Type.

China • Yunnan Province, Wenshan Zhuang and Miao Autonomous Prefecture, Malipo County, Xiajinchang Township, 23°10'45.1"N, 104°48'12.7"E, isolated from the root of forest-grown *P. notoginseng*, January 2025, Shu-Ying Yang, holotype HKAS 155214, ex-type culture KUNCC 11820.

##### Additional examined strains.

China • Yunnan Province, Wenshan Zhuang and Miao Autonomous Prefecture, Malipo County, Xiajinchang Township, 23°10'45.1"N, 104°48'12.7"E, isolated from the root of forest-grown *Panax notoginseng*, January 2025, Shu-Ying Yang, KUNCC 11829, KUNCC 11830, and KUNCC 11831.

##### GenBank accession numbers.

KUNCC 11820: *rpb2*: PZ417050; *tef1*: PZ417048.

##### Diagnosis.

Phylogenetically, *Tri. wenshanense* formed a distinct lineage closely related to *Tri. floccosum*. It differs from *Tri. floccosum* in having subglobose to broadly ellipsoidal conidia and lacking chlamydospores.

##### Description.

**Sexual morph**: Unknown. **Asexual morph**: Conidiophores with a distinct main axis; branches opposite or verticillate, arising at nearly right angles, forming a typical tree-like branching structure of *Trichoderma*. Phialides solitary or in whorls of 2–3, ampulliform, swollen at the base and gradually tapering towards the apex, (4.0–)4.3–6.5(–7.3) × (2.9–)3.1–4.5(–5.3) μm (av. 5.2 × 3.7 μm, *l/w* = 1.4, *n* = 44). Conidia subglobose to broadly ellipsoidal, pale green, smooth-walled, usually aggregated in clusters, (2.9–)3.0–4.2(–4.4) × (2.1–)2.2–3.1(–3.2) μm (av. 3.7 × 2.6 μm, *l/w* = 1.4, *n* = 32).

**Figure 4.**
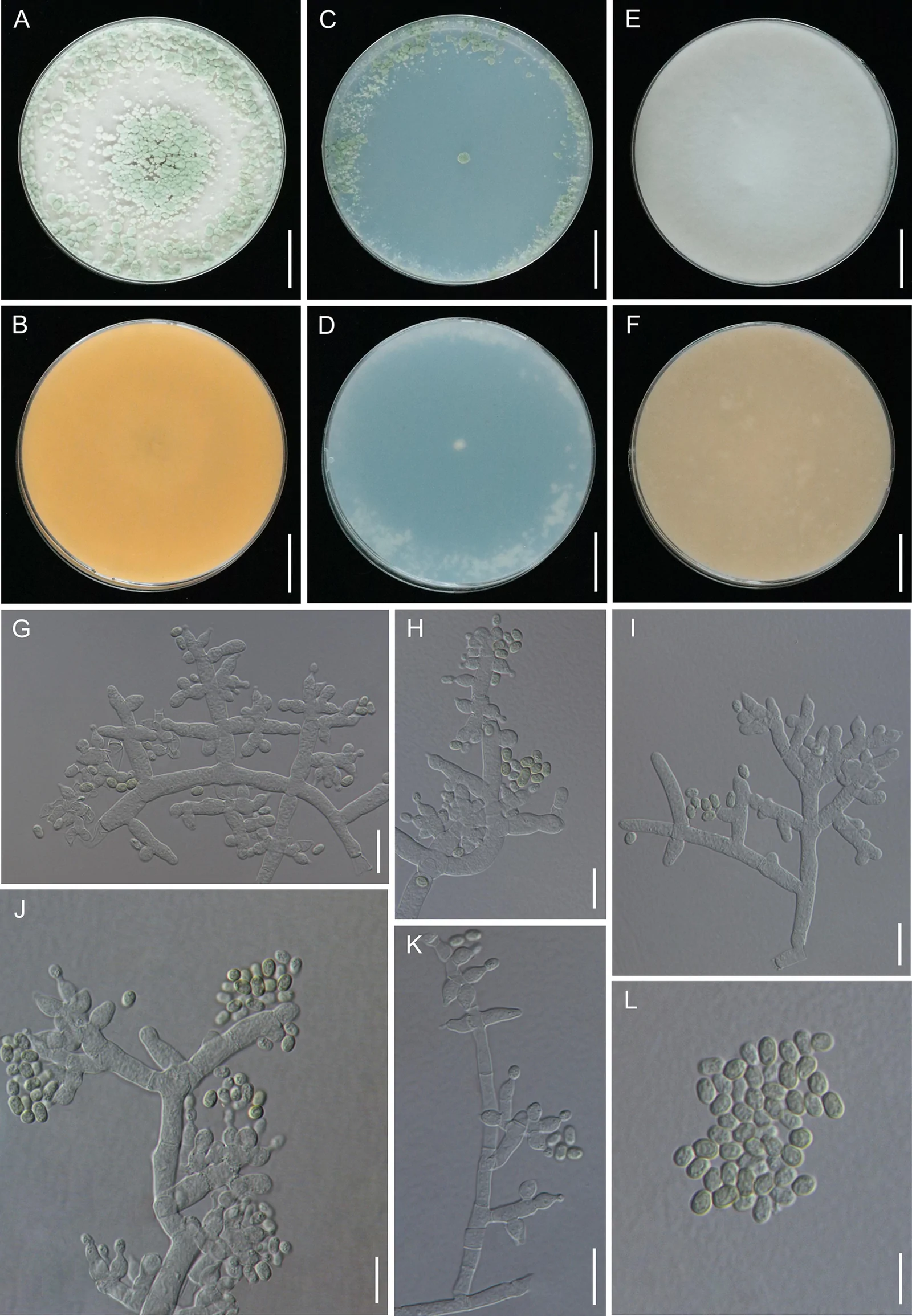
Morphological characteristics of *Trichoderma wenshanense* (ex-type culture KUNCC 11820). **A–F**. Colonies after 14 d at 25 °C; upper row, colony surfaces on PDA, SNA, and OA, respectively; lower row, colony reverses on the corresponding media; **G–K**. Conidiophores and phialides; **L**. Conidia. Scale bars: 10 mm (**A–F**); 10 μm (**G–L**).

##### Culture characteristics.

On PDA, colonies covering a 90 mm Petri dish after 7 d at 25 °C, white, mostly vegetative, with grayish-green conidiation beginning at the center; aerial mycelium dense, floccose to cottony; no diffusing pigment produced; odor indistinct. After 14 d, colonies white to pale green, with a green conidiation zone at the center and minute granular conidiation structures near the margin; reverse uniformly pale orange-yellow, without dark pigmentation or radiating grooves.

On SNA, colonies with only a thin layer of sparse white mycelium after 7 d at 25 °C, without visible conidiation; no diffusing pigment produced; odor indistinct. After 14 d, colonies covering a 90 mm Petri dish, with sparse, hyaline to white mycelium mainly distributed along the plate margin; a pale green, nearly circular conidiation zone ca. 0.4 cm in diam. formed at the inoculation point; margin with a green conidiation ring; conidiation abundant; no distinct concentric zones or diffusing pigment observed; reverse without obvious colony elevation or darkened areas, very pale whitish to slightly yellowish only at the inoculation point; odor indistinct.

On OA, colonies covering a 90 mm Petri dish after 7 d at 25 °C, white, without visible conidiation; no diffusing pigment produced; odor indistinct. After 14 d, colonies white to pale grayish white, with fluffy mycelium and sparse conidiation, without concentric zones; reverse without diffusing pigment; odor indistinct.

##### Distribution.

Currently known from Wenshan, Yunnan Province, China.

##### Notes.

Phylogenetically, *Tri. wenshanense* was closely related to *Tri. floccosum* (ex-type CBS 124372), differing from the latter by 3.6% (15/422 bp) in *tef1* and 1.3% (11/869 bp) in *rpb2*. Morphologically, it is distinguished from *Tri. floccosum* by its subglobose to broadly ellipsoidal conidia (vs. ellipsoidal), absence of chlamydospores (vs. abundant), and pale orange-yellow colony reverse on PDA (vs. pale yellow to yellowish brown) (Samuels et al. 2012). On OA, it produces sparse conidiation without concentric zones, whereas *Tri. floccosum* forms weak concentric zones with more developed conidiation. These morphological characters further distinguish *Tri. wenshanense* from other morphologically similar species, including *Tri. harzianum*, *Tri. viride*, *Tri. yunnanense*, and *Tri. virens* (Chaverri and Samuels 2002; Chaverri et al. 2003; Yu et al. 2007; Tozlu et al. 2018). The additional isolates examined in this study were genetically consistent with the ex-type strain and clustered together with it in the multilocus phylogeny, further supporting their assignment to *Tri. wenshanense*.

#### Clonostachys grandispora

Taxon classificationFungiHypocrealesBionectriaceae

S.Y. Yang, Y.B. Wang, J.P. Li & Z.W. Wang sp. nov.

AE8C76D2-DBE1-51E8-B69B-FEEFA051E1CF

Fungal Names: FN 573989

Fig. 5

##### Etymology.

The specific epithet “*grandispora*” refers to the relatively large conidia of this species, which are distinctly larger than those of closely related species in the genus.

##### Type.

China • Yunnan Province, Wenshan Zhuang and Miao Autonomous Prefecture, Malipo County, Xiajinchang Township, 23°10'45.1"N, 104°48'12.7"E, isolated from the root of forest-grown *Panax notoginseng*, January 2025, Shu-Ying Yang, holotype HKAS 155215, ex-type culture KUNCC 11821.

##### Additional examined strains.

China • Yunnan Province, Wenshan Zhuang and Miao Autonomous Prefecture, Malipo County, Xiajinchang Township, 23°10'45.1"N, 104°48'12.7"E, isolated from the root of forest-grown *Panax notoginseng*, January 2025, Shu-Ying Yang, KUNCC 11834.

##### GenBank accession numbers.

KUNCC 11821: ITS: PZ390434; LSU: PZ716387; *rpb2*: PZ417049; *tef1*: PZ417046; *tub2*: PZ417044.

##### Diagnosis.

Differs from the closely related *Clo. hongkongensis* by its markedly larger, ellipsoidal to short cylindrical conidia.

##### Description.

**Sexual morph**: Unknown. **Asexual morph**: Conidiophores erect, septate, with regular, simple, alternate branches. Phialides slenderly clavate to subcylindrical, solitary or in groups of 2–3, (9.2–)9.5–14.4(–14.6) × (2.1–)2.2–3.7(–3.8) μm (av. 11.7 × 3.0 μm, *l/w* = 4.0, *n* = 20). Conidia large, ellipsoidal to short cylindrical, with obtuse ends, hyaline, aseptate, smooth-walled, (7.2–)7.6–10.5(–11.7) × (2.0–)2.4–3.8(–4.0) μm (av. 9.4 × 2.9 μm, *l/w* = 3.3, *n* = 29), often aggregated in chains or heads at the apices of conidiogenous cells.

**Figure 5.**
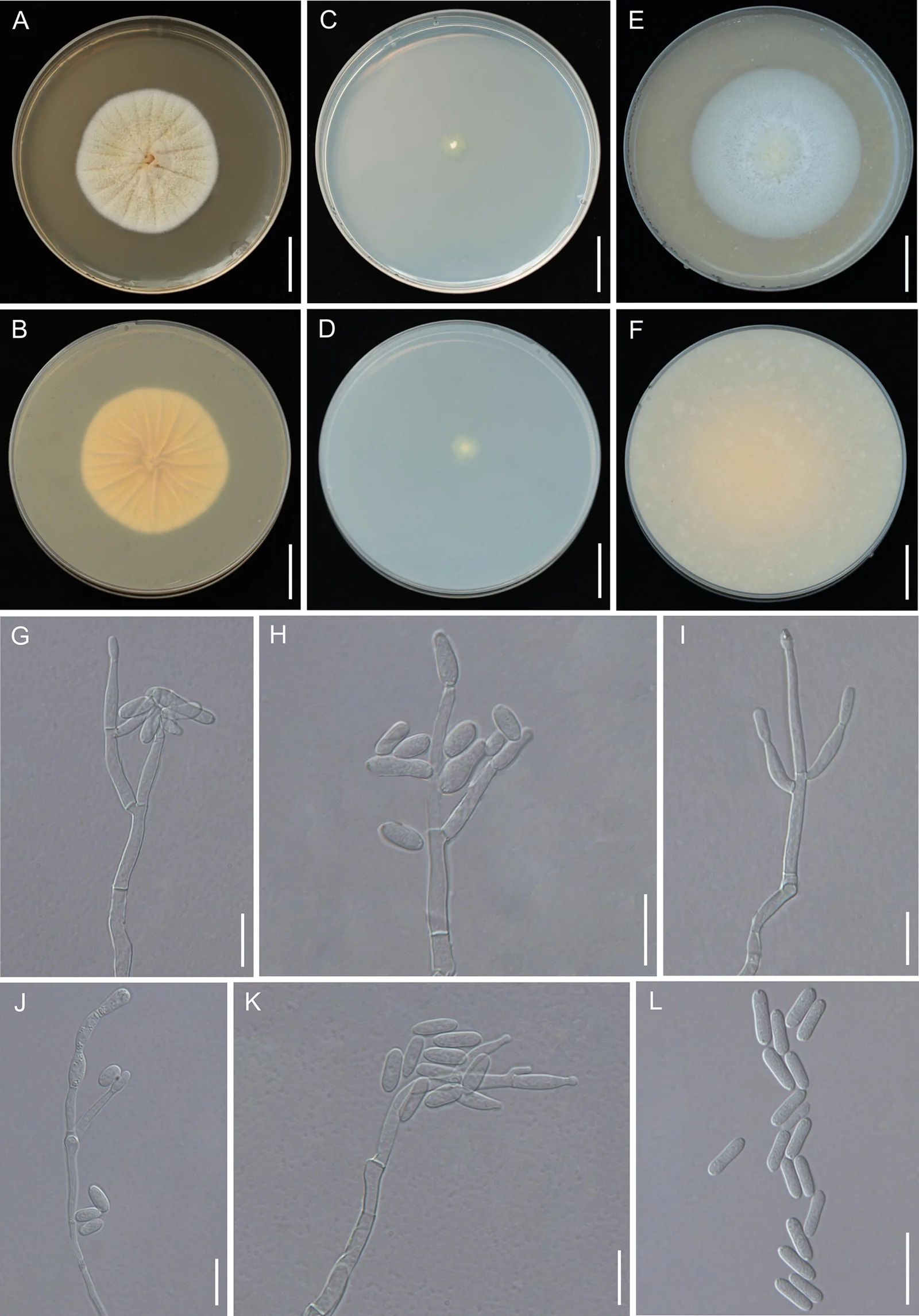
Morphological characteristics of *Clonostachys grandispora* (ex-type culture KUNCC 11821). **A–F**. Colonies after 14 d at 25 °C; upper row, colony surfaces on PDA, SNA, and OA, respectively; lower row, colony reverses on the corresponding media; **G–K**. Conidiophores and phialides. **L**. Conidia. Scale bars: 10 mm (**A–F**); 10 μm (**G–L**).

##### Culture characteristics.

On PDA, colonies reaching 2.2 cm diam. after 7 d at 25 °C, circular, white to pale cream, slightly pale yellow at the center; mycelium dense, fluffy, slightly raised at the center, with faint radiating folds; reverse pale orange, with radiating striations; odor indistinct. After 14 d, colonies reaching 5.1 cm diam., circular, pale yellowish brown to light brown, with distinct radiating folds, slightly raised at the center; mycelium thin and dense; reverse pale yellow to cream, with distinct radiating striations; pigment slightly penetrating into the medium, but without extensive diffusion.

On SNA, a minute colony ca. 0.8 cm in diam. formed at the inoculation point after 7 d at 25 °C, with a thin layer of sparse white mycelium; visible conidiation absent. After 14 d, the colony reached ca. 1.0 cm in diam. at the inoculation point and appeared as a small pale-yellow spot, without obvious mycelial extension or visible conidiation; reverse pale whitish to slightly yellowish only at the inoculation point, without pigment diffusion or discoloration of the medium; odor indistinct. Overall morphology differed little from that observed after 7 d.

On OA, colonies reaching 2.1 cm diam. after 7 d at 25 °C, circular, white to pale grayish white, without visible conidiation; reverse pale yellowish white, with very weak pigmentation; odor indistinct. After 14 d, colonies reaching 5.8 cm diam., circular, with an entire margin, pale cream-yellow at the center and milky white toward the periphery; mycelium velvety, without concentric zones or folds; no diffusing pigment produced; reverse pale yellowish white, with very weak pigmentation; odor indistinct.

##### Distribution.

Currently known from Wenshan, Yunnan Province, China.

##### Notes.

Phylogenetically, *Clo. grandispora* formed a distinct lineage sister to *Clo. hongkongensis* with strong support (100/1). It differed from *Clo. hongkongensis* (ex-type CBS 115291) by 0.8% (4/495 bp) in ITS, 0.6% (5/792 bp) in LSU, 5.8% (44/753 bp) in *rpb2*, 2.8% (23/809 bp) in *tef1*, and 2.4% (26/1064 bp) in *tub2*. Compared with *Clo. hongkongensis* CBS 116542, it differed by 0.8% (4/499 bp) in ITS, 0.6% (5/792 bp) in LSU, 5.8% (43/743 bp) in *rpb2*, and 2.8% (23/809 bp) in *tef1*. A comparable *tub2* sequence was unavailable for CBS 116542. Morphologically, *Clo. grandispora* is distinguished from *Clo. hongkongensis* by its markedly larger, ellipsoidal to short cylindrical conidia (av. 9.4 × 2.9 μm vs. 4.8 × 3.1 μm), whereas the conidia of *Clo. hongkongensis* are smaller and obovoid. Culturally, *Clo. grandispora* produces a pale yellow to cream colony reverse with distinct radiating striations on PDA, whereas *Clo. hongkongensis* has a pale yellow reverse without obvious radiating striations. On OA, *Clo. grandispora* develops velvety mycelium with a pale cream-yellow center and lacks concentric zones (Zhao et al. 2023). Thus, the combined molecular, morphological, and cultural evidence supports the recognition of *Clo. grandispora* as a distinct species. The additional isolate examined in this study showed morphological and molecular characteristics consistent with those of the ex-type strain and clustered together with it in the multilocus phylogeny, supporting its inclusion within *Clo. grandispora*.

#### Thelonectria chlamydosporia

Taxon classificationFungiHypocrealesNectriaceae

S.Y. Yang, Y.B. Wang, J.P. Li & Z.W. Wang sp. nov.

109A4A74-A3F5-53CA-A44E-D1774B51E983

Fungal Names: FN 573990

Fig. 6

##### Etymology.

The specific epithet “*chlamydosporia*” refers to the abundant production of chlamydospores in culture, which occur singly, in pairs, or in chains.

##### Type.

China • Yunnan Province, Wenshan Zhuang and Miao Autonomous Prefecture, Malipo County, Xiajinchang Township, 23°10'45.1"N, 104°48'12.7"E, isolated from the root of forest-grown *P. notoginseng*, January 2025, Shu-Ying Yang, holotype HKAS 155216, ex-type culture KUNCC 11822.

##### Additional examined strains.

China • Yunnan Province, Wenshan Zhuang and Miao Autonomous Prefecture, Malipo County, Xiajinchang Township, 23°10'45.1"N, 104°48'12.7"E, isolated from the root of forest-grown *Panax notoginseng*, January 2025, Shu-Ying Yang, KUNCC 11832, KUNCC 11833.

**Figure 6.**
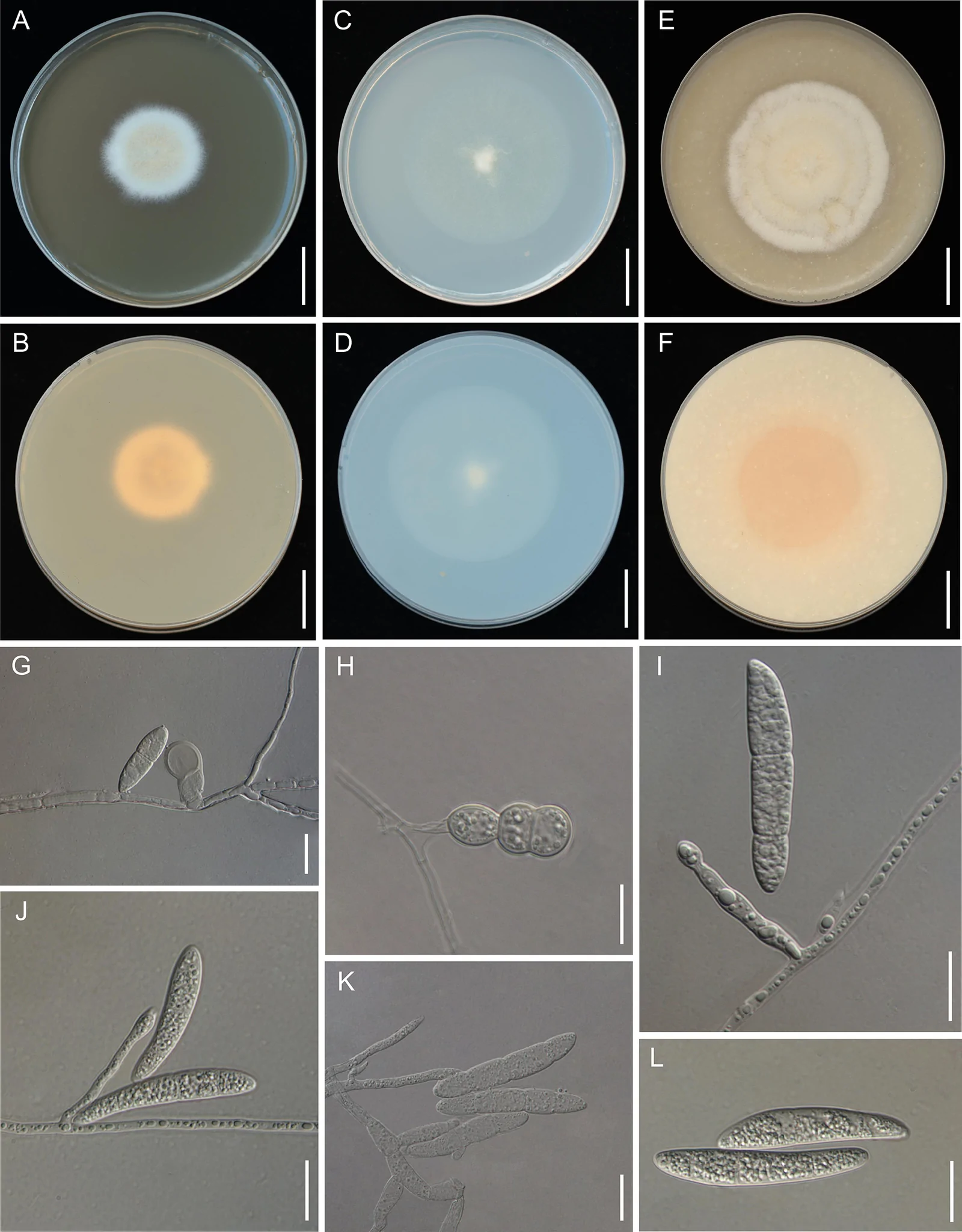
Morphological characteristics of *Thelonectria chlamydosporia* (ex-type culture KUNCC 11822). **A–F**. Colonies after 14 d at 25 °C; upper row, colony surfaces on PDA, SNA, and OA, respectively; lower row, colony reverses on the corresponding media; **G, I–L**. Phialides and conidia; **H**. Chlamydospores. Scale bars: 10 mm (**A–F**); 10 μm (**G–L**).

##### GenBank accession numbers.

KUNCC 11822: ITS: PZ390476; LSU: PZ390515; *rpb1*: PZ417042; *tef1*: PZ417047; *tub2*: PZ417045; *act*: PZ417043.

##### Diagnosis.

Differs from closely related species of *Thelonectria* by the abundant production of chlamydospores, which occur singly, in pairs, or in chains of 3–4; the closely related *The. blackeriella* species complex produces no or very few chlamydospores.

##### Description.

**Sexual morph**: Unknown. **Asexual morph**: Conidiophores solitary or sparsely branched, erect or slightly curved; branching simple, mostly monoaxial, occasionally dichotomous; intercalary or terminal chlamydospores also present on the branches. Phialides ampulliform to short cylindrical, solitary or in groups of 2–3, (14.3–)15.7–33.8(–34.4) × (2.6–)3.2–6.3(–6.6) μm (av. 24.7 × 4.7 μm, *l/w* = 5.7, *n* = 18). Conidia long cylindrical to fusiform, hyaline, smooth-walled, mostly aseptate, but occasionally 1–2-septate at maturity; cytoplasm containing numerous conspicuous granules, (28.8–)30.2–54.3(–55.6) × (6.5–)6.6–9.0(–9.2) μm (av. 42.3 × 7.7 μm, *l/w* = 5.6, *n* = 27), often borne singly or in groups of 2–3 at the apices of conidiogenous cells. Chlamydospores abundant, intercalary or terminal, globose to subglobose, smooth-walled, thick-walled, pale brown, solitary, in pairs or arranged in chains of 3–4, (5.6–)5.9–12.0(–12.7) × (5.2–)6.1–11.4(–11.5) μm (av. 8.7 × 8.9 μm, *l/w* = 1.0, *n* = 20).

##### Culture characteristics.

On PDA, colonies reaching 2.1 cm diam. after 7 d at 25 °C, white to pale grayish white; mycelium dense, velvety; margin entire, without radiating folds or obvious pigment diffusion; reverse pale orange; odor indistinct. After 14 d, colonies reaching 3.2 cm diam., circular, slightly raised at the center, pale orange-yellow; marginal mycelium white, entire, dense, velvety to cottony; reverse pale orange to light orange, slightly darker at the center; no diffusing pigment produced.

On SNA, colonies reaching 2.5 cm diam. after 7 d at 25 °C, with a thin layer of sparse white mycelium; mycelium flat and sparse, without visible conidiation. After 14 d, colonies reaching 5.6 cm diam., circular, with an extremely thin circular layer of white mycelium at the center; texture sparse and flat, showing radiating growth, with finely velvety mycelium visible to the naked eye; reverse colorless to very pale yellow, without pigment diffusion; odor indistinct.

On OA, colonies reaching 2.3 cm diam. after 7 d at 25 °C, white to pale grayish white, with dense mycelium; reverse pale orange; odor indistinct. After 14 d, colonies reaching 5.3 cm diam., circular, cottony and dense; surface pale yellow, with distinct concentric zones and powdery texture; reverse pale orange to light orange-red, with darker pigmentation at the center; odor indistinct.

##### Distribution.

Currently known from Wenshan, Yunnan Province, China.

##### Notes.

Phylogenetically, *The. chlamydosporia* was closely related to *The. blackeriella*. It differed from *The. blackeriella* (ex-type CBS 142200) by 5.2% (27/518 bp) in ITS, 3.6% (19/532 bp) in LSU, 1.6% (10/623 bp) in *rpb1*, 1.1% (6/567 bp) in *act*, and 3.4% (17/501 bp) in *tub2*. Morphologically, the new species is readily distinguished from these closely related taxa by its abundant production of chlamydospores, which are intercalary or terminal, globose to subglobose, smooth-walled, thick-walled, and pale brown, often solitary, in pairs, or arranged in chains of 3–4; in contrast, *The. blackeriella* produces no or very few chlamydospores under the same culture conditions. Additionally, the conidia of the new species are larger (av. 42.3 × 7.7 μm) and long cylindrical to fusiform, whereas those of *The. blackeriella* are distinctly smaller. On OA, the new species produces sparse conidiation, whereas abundant conidiation is reported in the closely related species (Salgado-Salazar et al. 2016). The additional isolates examined in this study shared morphological and molecular characteristics consistent with those of the ex-type strain and clustered together in the multilocus phylogeny, further supporting their assignment to *Thelonectria chlamydosporia*.

## Discussion

Endophytic fungi associated with medicinal plants have attracted increasing attention because of their ecological functions and potential applications in plant growth promotion, disease resistance, and secondary metabolite production. Specifically, endophytic fungi from *P. notoginseng* have shown potential in promoting plant growth and enhancing disease resistance (Chen et al. 2016). *Hypocreales* represents an important component of the endophytic fungal community associated with *P. notoginseng*. Strains assigned to *Trichoderma* and *Clonostachys* have previously been isolated from this host, and *Trichoderma* has also been frequently detected in healthy *P. notoginseng*. However, many recovered fungi were identified only at the genus level, and species-level taxonomic information remains limited (Zheng et al. 2017; Li et al. 2021; Lin et al. 2024). Consequently, species-level taxonomic information on *Trichoderma*, *Clonostachys*, and *Thelonectria* occurring as root endophytes of forest-grown *P. notoginseng* remains limited.

In this study, three new species, namely *Tri. wenshanense*, *Clo. grandispora*, and *The. chlamydosporia*, were described from endophytic fungal isolates associated with forest-grown *P. notoginseng*. These species belong to *Trichoderma (Hypocreaceae)*, *Clonostachys (Bionectriaceae)*, and *Thelonectria (Nectriaceae)*, respectively. The combination of diagnostic morphological characters, cultural characteristics, and multilocus phylogenetic evidence, together with the examination of additional conspecific strains, supports the recognition of these three taxa as distinct new species and clarifies their species-level taxonomic positions, contributing to the systematic understanding of hypocrealean endophytes associated with *P. notoginseng*.

From an ecological perspective, closely related species within the same genera as the three new taxa have been reported from diverse ecological niches. For example, *Tri. floccosum*, *Clo. hongkongensis*, and members of *Thelonectria* have been documented in previous taxonomic studies or from plant-associated habitats (Samuels et al. 2012; Salgado-Salazar et al. 2016; Zhao et al. 2023). These records suggest that the new taxa described here may also have potential ecological significance. However, their roles in the control of root rot diseases, plant growth promotion, or improvement of medicinal quality in *P. notoginseng* require further experimental evaluation.

Beyond their potential ecological roles, accurate species identification is essential for the effective exploration and utilization of endophytic fungal resources. Although numerous endophytic fungi have been isolated from *P. notoginseng* in previous studies, many strains have been reported without detailed species-level taxonomic resolution (Zhang and Li 2011; Zheng et al. 2017; Li et al. 2021; Lin et al. 2024). In this study, comprehensive morphological observations and multilocus phylogenetic analyses were performed for the three new species. During the isolation process, 188 endophytic fungal isolates were obtained from 23 *P. notoginseng* plants, including 70 isolates belonging to *Hypocreales*. Following further examination of the fungal collection, additional strains corresponding to the three newly described species were identified and incorporated into the revised analyses. These additional strains showed phylogenetic placement consistent with that of their corresponding type strains, providing additional evidence for evaluating intraspecific variation and supporting the recognition of *Tri. wenshanense*, *Clo. grandispora*, and *The. chlamydosporia* as distinct species. The three species represent only a subset of the *Hypocreales* diversity recovered from this host, suggesting that additional *Hypocreales* taxa associated with this host may warrant further investigation. The combination of multilocus phylogenetic relationships, nucleotide sequence divergence, diagnostic morphological characteristics, and cultural features provides a robust basis for species delimitation. Overall, this integrative taxonomic treatment provides an accurate and reliable taxonomic basis for subsequent functional studies. It further enriches the known species diversity of endophytic fungi associated with *P. notoginseng* and provides taxonomically characterized fungal resources for future studies on their potential roles in ecological cultivation and medicinal quality improvement of forest-grown *P. notoginseng*.

## Supplementary Material

XML Treatment for Trichoderma wenshanense

XML Treatment for Clonostachys grandispora

XML Treatment for Thelonectria chlamydosporia

## Additional information

### Artificial Intelligence (AI) use

The authors accept full responsibility for the content of the manuscript, including the disclosure of any use of AI.

No AI tools were used in the preparation of this manuscript.

### Author ORCIDs

S.-Y. Yang https://orcid.org/0009-0002-6230-0458

X.-Y. Feng https://orcid.org/0000-0003-0064-0940

C.-Y. Wei https://orcid.org/0009-0006-2582-4745

W.-B. Zeng https://orcid.org/0000-0003-0125-4099

Y.-B. Wang https://orcid.org/0000-0002-3305-9418

J.-P. Li https://orcid.org/0009-0005-6582-9762

Z.-W. Wang https://orcid.org/0000-0002-6091-5486

### Data availability

All of the data that support the findings of this study are available in the main text or Supplementary Information.

## Supplementary materials

Supplementary material 1 Taxa, strains, GenBank accession numbers, and references used in the phylogenetic analyses Data type xlsx https://binary.pensoft.net/file/1802959This dataset is made available under the Open Database License (http://opendatacommons.org/licenses/odbl/1.0/). The Open Database License (ODbL) is a license agreement intended to allow users to freely share, modify, and use this Dataset while maintaining this same freedom for others, provided that the original source and author(s) are credited. Shu-Ying Yang, Xiu-Yan Feng, Cui-Yuan Wei, Wen-Bo Zeng, Yuan-Bing Wang, Jian-Ping Li, Zhi-Wei Wang
